# The Physiological Action of Fatty Inunctions

**Published:** 1880-10-20

**Authors:** W. K. Harrison


### THE PHYSIOLOGICAL ACTION OF FATTY INUNCTIONS.

BY W. K. HARRISON, M.D.

The use of oily inunction in the treatment of disease has been in vogue from very early times. The anointing of the sick with oil is mentioned in the Bible and in many other ancient writings, and is advocated by medical authors from those ancient days down to the present time. From observation and inquiry, I am inclined to believe that fatty inunction is practiced more by eclectics than by other schools of medicine. It is undoubtedly attended with good results, and must be based upon sound physiological principles. In the febrile conditions of children, where there is a harsh, dry skin, loss of appetite, rapid pulse, great thirst, etc., fatty inunctions are often followed by a rapid amelioration of the symptoms, and in two or three hours' time, the skin is found to be cooled and soft, and a quiet, comfortable sleep is the result. Experience teaches us to expect great benefit from the external use of fats in scarlet fever and measles, with high temperatures, and these are the diseases in which they are commonly recommended, but they are not a whit less useful in other febrile conditions. In the summer diseases of children, where there is fever accompanied with disturbances of the alimentary canal, fatty inunction not only mitigates the fever, but also lessens intestinal irritation in a marked degree.

From actual experiment with the clinical thermometer, I am convinced that the external use of fat alone will often cause a reduction of from one to three degrees in temperature.

This reduction of temperature may be explained upon physiological grounds. Dr. Routh says, in "Infant feeding," there is probably in all animals, particularly young animals, a certain amount of cutaneous respiration, *i. e.*, some action between the oxygen of the atmosphere and the capillaries of the skin. The way in which this process is carried on, I do not presume to explain. If, however, this external communication with the oxygen of the air be cut off, the temperature falls several degrees. Becquerel and Breschet, in "Carpenter's physiology," found that the temperature of rabbits, which had been first shaved and then covered with varnish, fell in an hour from 100° to 76°, and in one instance to 69½°. An inunction of fat cuts off the oxygen of the air less perfectly, and the fall in temperature is less marked, yet its antipyretic action is decided, and may be utilized with great benefit in cases where remedies are not well received by the stomach, or in conjunction with such remedies. A therapeutic measure so simple, rational and harmless, is worthy of more extended use.
—*Chicago Med. Times.*

